# Identification of DHA-23, a novel plasmid-mediated and inducible AmpC beta-lactamase from Enterobacteriaceae in Northern Taiwan

**DOI:** 10.3389/fmicb.2015.00436

**Published:** 2015-05-06

**Authors:** Wen-Shyang Hsieh, Nai-Yu Wang, Jou-An Feng, Li-Chuan Weng, Hsueh-Hsia Wu

**Affiliations:** ^1^Department of Laboratory Medicine, Taipei Medical University – Shuang Ho Hospital, New Taipei CityTaiwan; ^2^School of Medical Laboratory Science and Biotechnology, Taipei Medical University, TaipeiTaiwan; ^3^Graduate Institute of Biomedical Informatics, Taipei Medical University, TaipeiTaiwan; ^4^Department of Medical Research, Mackay Memorial Hospital, New Taipei CityTaiwan; ^5^Department of Laboratory Medicine, Taipei Medical University Hospital, TaipeiTaiwan

**Keywords:** AmpC beta-lactamase, Enterobacteriaceae, antimicrobial resistance epidemiology

## Abstract

**Objectives:** AmpC β-lactamases are classified as Amber Class C and Bush Group 1. AmpC β-lactamases can hydrolyze broad and extended-spectrum cephalosporins, and are not inhibited by β-lactamase inhibitors such as clavulanic acid. This study was conducted to identify DHA-23, a novel plasmid-mediated and inducible AmpC β-lactamase obtained from Enterobacteriaceae.

**Methods:** A total of 210 carbapenem-resistant Enterobacteriaceae isolates were collected from a medical center (comprising two branches) in Northern Taiwan during 2009–2012. AmpC β-lactamase genes were analyzed through a polymerase chain reaction using plasmid DNA templates and gene sequencing. The genetic relationships of the isolates were typed using pulsed-field gel electrophoresis following the digestion of intact genomic DNA by using *Xba*I.

**Results:** Three enterobacterial isolates (one *Escherichia coli* and two *Klebsiella pneumoniae*) were obtained from three hospitalized patients. All three isolates were resistant or intermediately susceptible to all β-lactams, and exhibited reduced susceptibility to carbapenems. These three isolates expressed a novel AmpC β-lactamase, designated DHA-23, approved by the curators of the Lahey website. DHA-23 differs from DHA-1 and DHA-6 by one amino acid substitution (Ser245Ala), exhibiting three amino acid changes compared with DHA-7 and DHA-*Morganella morganii*; three amino acid changes compared with DHA-3; four amino acid changes compared with DHA-5; and eight amino acid changes compared with DHA-2 (>97% identity). This AmpC β-lactamase is inducible using a system involving *ampR*.

**Conclusion:** This is the first report to address DHA-23, a novel AmpC β-lactamase. DHA-type β-lactamases are continuous threat in Taiwan.

## Introduction

AmpC enzymes are located in the bacterial periplasm, with the exception of the AmpC β-lactamase of *Psychrobacter immobilis*, which is secreted mainly into the external medium (Feller et al., 1997). They are active on cephalosporins, cephamycins (such as cefoxitin), oxyiminocephalosporins (such as ceftazidime and cefotaxime), and monobactams (such as aztreonam). AmpC β-lactamases are classified according to their Amber molecular structure as belonging to Class C, whereas according to function, they are classified into Group 1 (Bush and Jacoby, 2010). The sequence of the *ampC* gene differed from the sequence of penicillinase-type β-lactamases such as TEM-1 but similarly, had serine at its active site (Knott-Hunziker et al., 1982). AmpC β-lactamases, in contrast to extended-spectrum β-lactamases (ESBLs), can hydrolyze broad and extended-spectrum cephalosporins, and are not inhibited by β-lactamase inhibitors such as clavulanic acid. AmpC β-lactamases are assumed to be chromosomally mediated; however, *Klebsiella pneumoniae*, *K. oxytoca*, *Proteus mirabilis*, and *Salmonella* sp. lack a chromosomal *bla*_AmpC_ gene (Bergstrom et al., 1983; Bauernfeind et al., 1989). Described plasmid-mediated *AmpC* genes in a *K. pneumoniae* isolate from South Korea. There are various types of plasmid-mediated AmpC β-lactamases: CMY, MIR, MOX, LAT, FOX, DHA, ACT, ACC, and CFE (Jacoby, 2009). AmpR is a member of the LysR transcriptional regulator family. During normal growth, in the absence of β-lactam as an inducer, the AmpR regulator binds with a peptidoglycan precursor uridine pyrophosphoryl-*N*-acetyl muramyl-L-alanyl-D-glutamyl*meso*-diaminopimelic acid-D-alanyl-D-alanine (UDP-*N*-acetylmuramic acid peptide). AmpR- UDP-*N*-acetylmuramic acid peptide complex binds to the operator site between the *ampC* and *ampR* structural genes, leading to the repression of *ampC* expression. Displacement of the UDP-*N*-acetylmuramic acid peptides signals a conformational change in AmpR, which activates the transcription of *ampC*. AmpR mutations are less common but can also result in high-constitutive or hyperinducible phenotypes (Kaneko et al., 2005). Resistance caused by plasmid-mediated AmpC β-lactamase is less common than the production of ESBLs, but may be more difficult to detect. The purpose of this study was to identify DHA-23, a novel plasmid-mediated and inducible AmpC β-lactamase identified in clinical enterobacterial isolates that were obtained from a hospital in Northern Taiwan.

## Materials and Methods

### Isolates and Data Collection

A total of 210 carbapenem-resistant Enterobacteriaceae isolates were collected from a medical center (comprising two branches) in Northern Taiwan from 2009 to 2012, including 100 *K. pneumoniae*, 53 *Escherichia coli*, 41 *Enterobacter cloacae*, and 16 other isolates (one *K. oxytoca*, one *Citrobacter freundii*, two *Providencia rettgeri*, eight *Serratia* sp., and four *E. aerogenes*). The isolates were identified using the VITEK2 system (bioMérieux Vitek Systems Inc., Hazelwood, MO, USA).

### Polymrase Chain Reaction Detection of Carbapenemase Genes and Insertion Sequences

The carbapenemase-encoding genes were detected using polymerase chain reaction (PCR) methods as previously suggested by Woodford et al. (2006) and Ellington et al. (2007). All primers used in this study, such as *bla*_TEM_-type, *bla*_SHV_-type, *bla*_CTX-M_-type, *bla*_CMY_-type, and *bla*_DHA_-type primers, were as described previously (Tenover et al., 1995; Chung et al., 2011; Qi et al., 2011; Huang et al., 2012). Sequence similarity searches were conducted using the BLAST program^1^.

### Pulsed-Field Gel Electrophoresis

The isolates were compared using pulsed-field gel electrophoresis (PFGE; Seifert et al., 2005) following the digestion of intact genomic DNA by using *Xba*I (Biolabs, UK). The *Xba*I restriction profiles were initially compared using visual inspection according to the criteria of Tenover et al. (1995). A computer-assisted analysis was performed using BioNumerics (Applied Maths, Sint-Martens-Latem, Belgium) software.

### Conjugation and Electrotransformation Experiments

Plasmid DNAs were extracted using the Qiagen Plasmid Purification Midi Kits (Qiagen, Courtaboeuf, France). Plasmid conjugation experiments were performed using *E. coli* DH5α as the recipient (Poirel et al., 1999b). Transconjugants were selected on Luria–Bertani agar plates supplemented with sodium azide (100 mg/L) and cefotaxime (2 mg/L). In addition, cefoxitin (8 mg/L) was added to prevent the selection of ESBL-producing transconjugants. Electrotransformants were selected on agar containing cefotaxime (2 mg/L) or cefoxitin (8 mg/L).

### Minimum Inhibitory Concentrations

Minimum inhibitory concentrations (MICs) were determined using the agar dilution method, which was repeated twice for each sample. MICs of tested antibiotics were interpreted according to Clinical and Laboratory Standards Institute guidelines (M7–A9, CLSI, 2012). Antibiotics were purchased from Sigma–Aldrich (St. Louis, MO, USA). Quality control was assured by testing *Staphylococcus aureus* ATCC 29213, *E. coli* ATCC25922, and *Pseudomonas aeruginosa* ATCC 27853.

## Results

### Novel AmpC β-Lactamase Discovered in Three Enterobacterial Isolates

**FIGURE 1:**
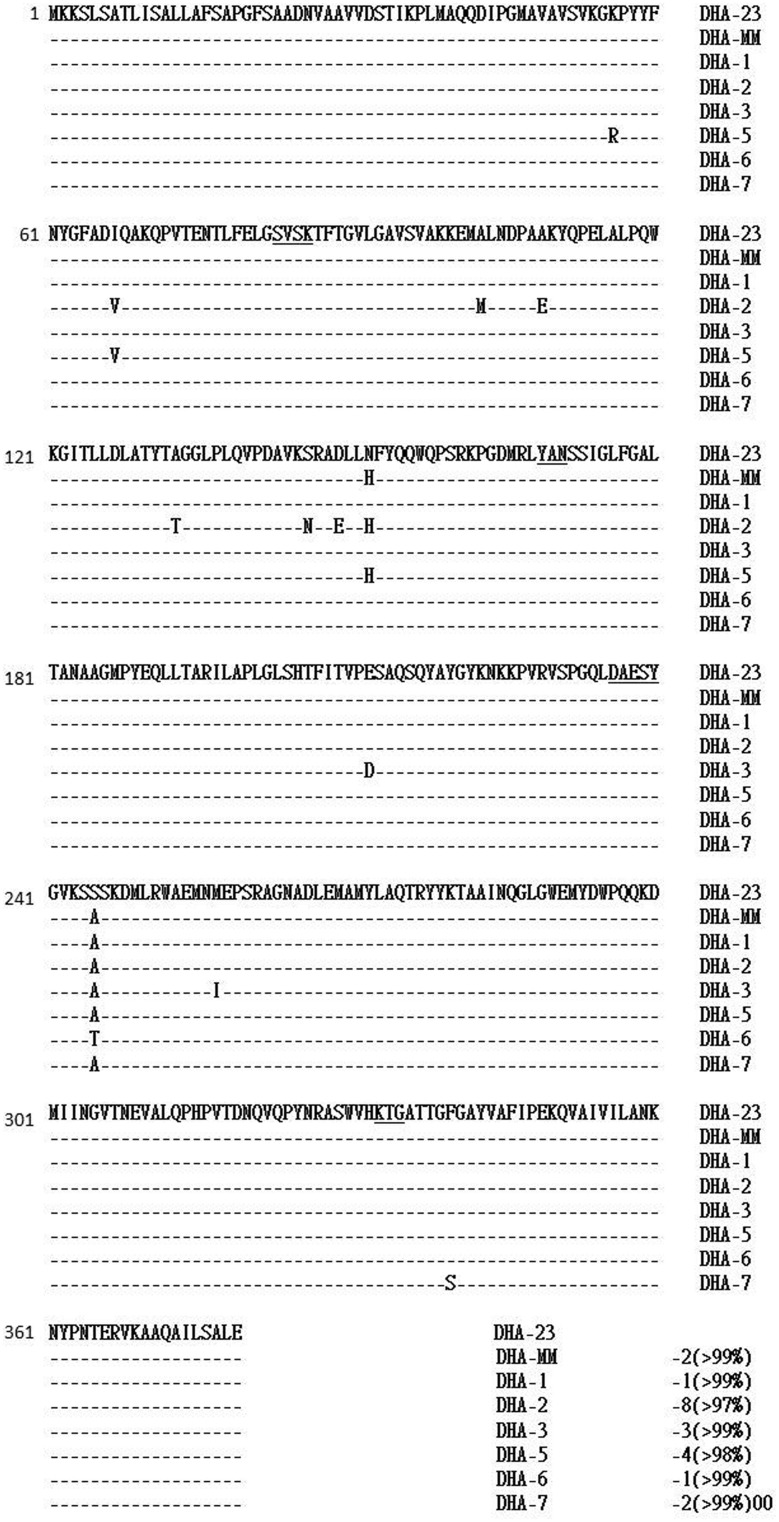
**Alignment of the deduced amino-acid sequences of DHA-23 with those of other DHA type AmpC enzymes**. Identical amino-acids are marked with dashes. The underlined amino-acids are those that may be involved in the catalytic site of these AmpC enzymes, including the β-lactamase active site S–V–S–K and the conserved triad K–T–G. DHA-MM, DHA-*Morganella morganii.*

One *E. coli* (EC56) and two *K. pneumoniae* (KP11 and KP19) isolates that exhibited resistance to cefoxitin, cefotaxime, and ceftazidime were isolated from 3 adult patients hospitalized in Northern Taiwan (**Table 1**). We had the designated DHA-23 approved by the curators of the Lahey website ^2^. Using specific primers for *bla*_DHA-1_, we obtained PCR fragments from plasmid DNA preparations of *E. coli* EC56 and *K. pneumoniae* KP11 and KP19. The deduced amino-acid sequence (**Figure 1**) indicated that DHA-23 exhibited only one amino-acid change compared with DHA-1 and DHA-6 (Ser245Ala), two amino acid changes compared with DHA-7 and DHA-*Morganella morganii* (DHA-MM; Barnaud et al., 1998), three amino acid changes compared with DHA-3 (Wu et al., 2005), four amino acid changes compared with DHA-5, and eight amino acid changes compared with DHA-2 (>97% identity; Fortineau et al., 2001).

**Table 1:** Minimum inhibitory concentrations (MICs) of three DHA-23 carrying enterobacterial isolates to antimicrobial agents.

Antimicrobial agent	MIC (mg/L)		
	KP11*	KP19*	EC56*
Ampicillin	≥32	≥32	≥32
Cefazolin	≥64	≥64	≥64
Cefoxitin	≥64	≥64	≥64
Cefuroxime	≥64	≥64	≥64
Cefotaxime	128	128	128
Ceftazidime	64	32	128
Flomoxef	≥64	≥64	≥64
Cefpirome	16	16	≥64
Ciprofloxacin	≥4	≥4	≥4
Moxifloxacin	≥8	≥8	≥8
Piperacillin-tazobactam	≥128	≥128	≥128
Trimethoprim-sulfamethoxazole	≥320	≥320	≥320
Amikacin	≥64	≥64	≥64
Gentamicin	≥16	≥16	≥16
Tigecycline	4	2	≤0.5
Colistin	≤0.5	≥16	≤0.5
Ertapenem	2	2	4
Meropenem	0.12	0.12	0.12
Imipenem	2	2	1
Doripenem	0.5	0.25	0.06

*KP11 and KP19 expressed TEM-1, SHV-11, CTX-M-14, and DHA-23, EC56 expressed TEM-1, CTX-M-14, CMY-2, and DHA-23.

### *ampR* Gene Found Immediately Upstream from the *ampC* Gene

**FIGURE 2:**
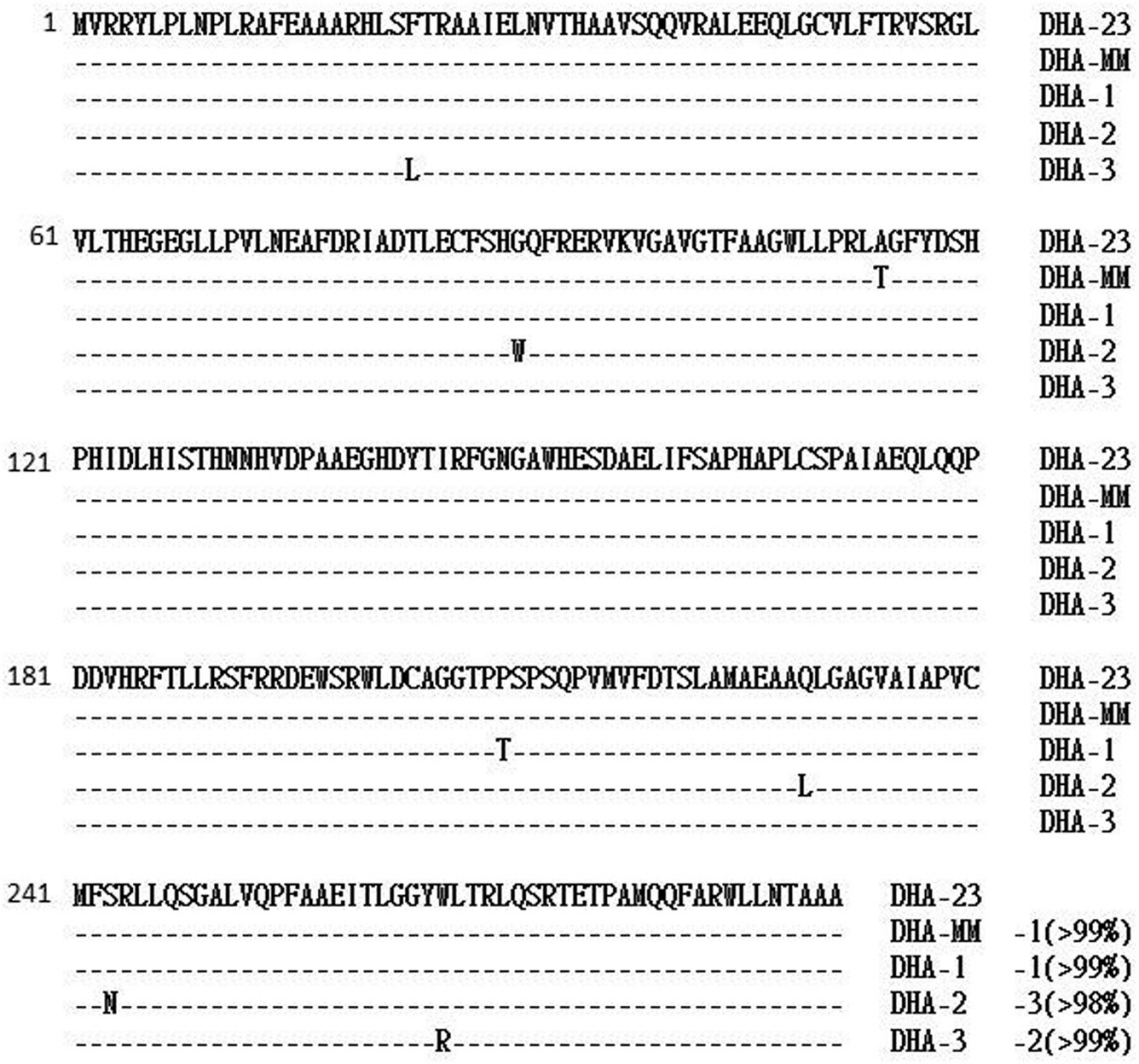
**Alignment of the deduced amino-acid sequences of the AmpR protein of DHA-23 with those of other DHA type AmpC enzymes**.

**FIGURE 3:**
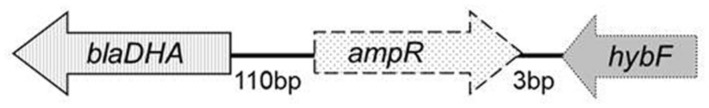
**Genetic environment of *bla*DHA-23**.

The 110-bp intercistronic region of *ampC* and *ampR* contained the promoter sequences for *ampC* and *ampR* expression. This region of *bla*_DHA-23_ was identical to the corresponding region of *bla*_DHA-1_ (Barnaud et al., 1998) and was 97.3% identical to that in the chromosome of *M. morganii* (Poirel et al., 1999a). The *ampR* gene had an overlapping and divergently oriented promoter, as described for other *ampC–ampR* regulatory systems (Bennett and Chopra, 1993). Comparing the deduced amino-acid sequences of the AmpR of DHA-23 with those of DHA-1 and *M. morganii* indicated >99% identity, with only one amino-acid change in each case (**Figure 2**). A comparison of the AmpR of DHA-2 or the AmpR of DHA-3 revealed that they were at least >98% similar, exhibiting two and three amino acid changes, respectively. Similar to DHA-1, DHA-2, and DHA-3 (Barnaud et al., 1998; Fortineau et al., 2001; Wu et al., 2005), the sequences surrounding *bla*_DHA_ showed that *hybF* was found upstream from the *ampC* gene in DHA-1, DHA-2, and DHA-3, whereas *orf-1* was found downstream from the *ampC* gene only in DHA-1 (Barnaud et al., 1998). Subsequently, *hybF* and *orf-1* were investigated in the sequences surrounding *bla*_DHA-23_. The *hybF* was identified downstream of *ampR*, but *orf-1* was not detected (**Figure 3**). The *hybF* was coded for HybF and shared sequence homologies with hydrogenase subunits of *E. coli* (Menon et al., 1994). Although *ampR* and *bla*_DHA_ were mobilized from the chromosome of *M. morganii* into a complex of In6–In7–*sulI*-type class-1 integrons in a strain of *Salmonella enterica* serovar Enteritidis (Verdet et al., 2000), an integron carrying *bla*_DHA-23_ was not detected in *E. coli* EC56 through PCR experiments employing 5′-CS and 3′-CS specific primers of class one integrons.

### Clavulanic Acid Acts as An Inducer of DHA-23

*E. coli* EC56 contained 4 β-lactamases that corresponded to TEM-1, CTX-M-14, CMY-2, and DHA-23. *K. pneumoniae* KP11 and KP19 both contained β-lactamases that corresponded to TEM-1, SHV-11, CTX-M-14, and DHA-23. The genotyping of isolates was conducted using PFGE by using *Xba*I digestion. The results indicated that these two *K. pneumoniae* isolates had similar pulsotypes. Conjugation experiments were subsequently conducted, regardless of whether cefoxitin was used as a selecting agent, and the transfer of a plasmid coding the DHA-23 cephalosporinase into *E. coli* DH5α was not observed. The data suggested that the *bla*_DHA-23_ plasmid is not self-transferable. Electroporation experiments were then performed using plasmid DNA preparation of *E. coli* EC56 and *K. pneumoniae* KP11 and KP19. Amplification and gene sequencing were used to analyze the plasmid DNA, and the results revealed that the *E. coli* DH5α (pEC56) transformants contained two β-lactamases, TEM-1 and DHA-23. The *E. coli* DH5α (pKP11) and *E. coli* DH5α (pKP19) transformants contained only one β-lactamase, TEM-1. However, repeated electrotransformation experiments failed to obtain transformants carrying DHA-23 from KP11 and KP19. The MIC of ceftazidime (128 mg/L) for *E. coli* EC56 was not reduced by clavulanic acid. However, the MICs of cefotaxime (0.12 mg/L) and ceftazidime (1.0 mg/L) for *E. coli* DH5α (pEC56) increased in the presence of clavulanic acid, suggesting that clavulanic acid may act as an inducer of DHA-23 (**Table 2**).

**Table 2:** Minimum inhibitory concentrations of β-lactams for clinical isolate *Klebsiella pneumoniae* KP19, *Escherichia coli* EC56, electroporant *E. coli* DH5α (pPK19), *E. coli* DH5α (pEC56), and reference strain *E. coli* DH5α.

Antimicrobial agent	MIC (mg/L)				
	KP19	EC56	*E. coli* DH5α (pKP19)*^#^*	*E. coli* DH5α (pEC56)^†^	*E. coli* DH5α
Ampicillin	≥32	≥32	≥32	≥32	≤2
Cefoxitin	≥64	≥64	≤4	32	≤4
Cefotaxime	128	128	0.06	0.12	0.03
Ceftazidime	32	128	0.25	1	0.25
Ceftazidime-clavulanate*	32	≥128	0.25	2	0.25
Cefpirome	16	≥64	≤1	≤1	≤1
Ciprofloxacin	≥4	≥4	≤0.25	≤0.25	≤0.25
Ertapenem	2	4	≤0.5	≤0.5	≤0.5
Imipenem	2	1	≤1	≤1	≤1

*Clavulanate was tested at a fixed concentration of 4 mg/L.

*^#^*E. coli* DH5α (pKP19) expressed the plasmid-mediated TEM-1from *K. pneumoniae* 19.*

*^†^*E. coli* DH5α (pEC56) expressed the plasmid-mediated TEM-1 and DHA-23 from *E. coli* 56.*

## Discussion

This study presented a novel plasmid-mediated and inducible AmpC β-lactamase obtained from Enterobacteriaceae, designated DHA-23 and approved by the curators of the Lahey website. There are currently 22 DHA variants listed on the site, most of which are assigned with no reference to the sequence.

In **Table 1**, MICs of the DHA-23 carrying enterobacterial isolates *E. coli* EC56 and *K. pneumoniae* KP11 and KP19 to antimicrobial agents show that cephamycins (such as cefoxitin) are not hydrolyzed by ESBLs, but are hydrolyzed by associated AmpC β-lactamase. Class-C enzymes hydrolyze cephamycins but do not hydrolyze extended-spectrum cephalosporins effectively. DHA-23 showed that the MICs did not substantially increase for cefotaxime and ceftazidime when DHA-23 was present in the transformants, whereas the MICs of cefoxitin increased to 32 mg/L (**Table 2**). Resistance to the carbapenems was variable. All strains were fully susceptible to meropenem and doripenem (**Table 2**). In general, carbapenems are regarded as the preferred agent for treatment. However, the production of AmpC β-lactamase significantly increased the MICs of carbapenems was reported by Bradford et al. with the ACT-1 β-lactamase (Bradford et al., 1997), and by Lee et al. (2010) with the DHA-1 β-lactamase. Based on the recombinant experiments, Martinez-Martinez et al. (1999) demonstrated that MICs of carbapenems increased significantly in the recombinant *K. pneumoniae* strain harboring over expressing AmpC β-lactamase and loss of porins. They proposed that the spread of strains that express the plasmid-mediated AmpC β-lactamases and lack porins may create serious therapeutic problems in the future. Furthermore, proteomic investigation of the inner-membrane fraction of carbapenem-resistant strain of *Acinetobacter baumannii* supported a model for the importance of upregulated AmpC β-lactamases and down-regulated OmpW production in the mediation of carbapenem resistance in *A. baumannii* (Tiwari et al., 2012).

Enterobacteriaceae isolates producing a DHA-1-like enzyme have been identified previously in Taiwan (Yan et al., 2002; Yu et al., 2004; Wu et al., 2005). We report that DHA-type β-lactamases remain a threat in this country. Further nationwide surveillance should be conducted, antibiotic stewardship should be advocated, and strict infection control measures should be enforced.

## Conflict of Interest Statement

The authors declare that the research was conducted in the absence of any commercial or financial relationships that could be construed as a potential conflict of interest.

## Footnotes

1 http://www.lahey.org/Studies/

2 http://www.ncbi.nlm.nih.gov/blast
